# The Role of Ohmic Heating in Tailoring Pea Protein Functionality

**DOI:** 10.3390/gels12010050

**Published:** 2026-01-02

**Authors:** Zita Avelar, Luís Loureiro, Ana Catarina Leite, António A. Vicente, Rui M. Rodrigues

**Affiliations:** 1 Centre of Biological Engineering (CEB), University of Minho, Campus de Gualtar, 4710-057 Braga, Portugal; 2 LABBELS—Associate Laboratory, 4710-057 Braga/Guimarães, Portugal

**Keywords:** emerging technologies, protein functionality, foaming capacity, foam stability, emulsifying capacity, cold-set gelation

## Abstract

Plant-derived proteins have been growing in interest for the design of innovative foods and ingredients following the trend of animal protein substitution. These proteins display interesting functional properties, including emulsifying, foaming, and gelling capacity. Unfortunately, commercially available plant protein ingredients often present limited functionality due to the modifications induced during production. In this study, ohmic heating (OH) was evaluated as a physical modification strategy to enhance the functionality of commercial pea protein (PP). PP dispersions were subjected to OH at 100 °C, 130 °C, and 150 °C, and their physicochemical, foaming, emulsifying, and gelling properties were assessed. OH processing significantly reduced mean particle size, with the surface-area weighted diameter (D(3,2)) decreasing from approximately 76.1 µm in untreated PP to 56.5, 31.1, and 10.6 µm after OH at 100, 130, and 150 °C, respectively. These structural changes resulted in a clear improvement in foaming performance, with foaming capacity increasing by approximately 40% compared to the control, while all foams remained stable for at least 60 min. In contrast, emulsifying activity showed no substantial enhancement. Cold-set gels prepared from OH-treated PP exhibited significantly altered rheological behavior, characterized by lower complex modulus values (G* ≈ 0.8–5.4 kPa at 1 Hz) compared to the untreated PP gel (≈25.2 kPa), indicating the formation of softer yet more homogeneous gel networks. Overall, the results demonstrate that OH is an effective tool to tailor the functional properties of commercial pea protein, particularly by enhancing foaming performance and modulating gel structure, supporting its potential application in the development of novel plant-based food products.

## 1. Introduction

The demand for plant-based proteins is quickly rising due to the increasing pressure of population growth and weakening of climatic conditions [1]. Pea protein (PP) has been gaining increasing interest due to a well-balanced amino acid ratio, low allergenicity, and the possibility of growing in mild climates [2]. Its commercial availability, coupled with the previous attributes, makes this protein a good option for utilization in a broad range of food applications [3].

The recent emphasis on plant proteins has been their use for meat substitution. Yet, their functionality capacity, including emulsification, foaming, and gelling capacity, enable them to be used in several other applications [4,5]. Unfortunately, while laboratory isolated PP often displays similar functional properties as soy protein, commercially available PP often present limited functionality, which reduces its usage. Indeed, harsher processing conditions, including alkaline extraction, isoelectric precipitation, and spray drying, are usually applied for commercial PP, causing protein denaturation and aggregation, which often compromises its technological–functional properties [6,7,8].

Therefore, inexpensive and nonchemical treatments for improving the functionality of these products is highly desirable. Protein modification by physical methods has been gaining traction due to their effectiveness and absence of exogenous substances [9]. Furthermore, these methods have high feasibility and potential for industrial implementation [10]. In this context, emerging processing technologies, such as ohmic heating (OH), aligning with the global demand of environmentally friendly and highly efficient technologies [11].

OH is a processing technology that used moderate electric fields (MEF) to generate heat [12], being a recognized as a successful alternative to conventional processing by improving shelf life and food quality. Additionally, OH has demonstrated the capacity to interfere with proteins’ structure, modulating their functionality [13,14]. Despite the existing studies of OH effects in protein structure, the use of this technology as a physical modification tool in commercial vegetal proteins and, specifically, PP remains scarce. Chen et al., 2022 used OH to induce physicochemical changes of pea protein isolate, resulting in gels with higher water holding capacity and more uniform microstructure [13]. In a recent study, the authors have reported that processing of commercial PP by OH at temperatures between 90 °C and 150 °C resulted in substantial solubility enhancement [15]. It was found that all treatments resulted in enhanced solubility (from 63% up to 240%) of a commercial PP sample. The solubility increase was attributed to the dissociation of the insoluble aggregates present in the commercial PP, and structural analysis revealed changes associated with the application of OH, causing a secondar structure rearrangement, rather than unfolding. Moreover, at higher treatment temperatures, OH application may have caused a partial protein hydrolysis, resulting on the formation of mainly small soluble aggregates and peptide fragments. Even though this work demonstrated the potential of OH as a physical modification technique, its impact on the functional performance of pea protein systems remains to be clarified.

Building upon the author’s previous research, the objective of this work was to explore the potential use of OH to enhance the functional properties of a commercial PP with limited functionality. For this purpose, PP dispersions were subjected to OH under previously unexplored conditions (100 °C, 130 °C, and 150 °C). Then, their foaming and emulsifying performances were evaluated, as well as the rheological properties of the gels obtained from a selected cold-set gelation process.

## 2. Results and Discussion

### 2.1. Particle Size Distribution and Rheological Properties

**Figure 1:**
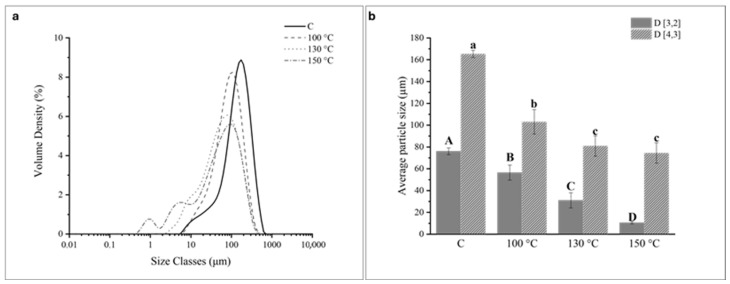
(**a**) Particle size distribution and (**b**) mean particle diameters D (3,2) and D (4,3) of control (C) and ohmic heating (OH)-treated pea protein (PP) dispersions. (**b**) For each column, different capital letters correspond to statistically significant differences (*p* < 0.05) relative to the D( 3,2) and different lower-case letters correspond to statistically significant differences (*p* < 0.05) relative to the D (4,3).

The particle size distribution of PP dispersions was accessed in control samples, not submitted to any treatment (C), and processed samples by OH (at 100 °C, 130 °C, and 150 °C). The results are shown in Figure 1a.

The C sample displayed a wide monomodal peak, comprehending that extended from around 6 to 700 μm. After the application of OH at 100 °C, the distribution curve shifted to the left and maintained the monomodal peak from around 6 to 500 μm. The increase of treatment temperature led to a progressive shift to the left, and at the highest treatment temperature tested (i.e., 150 °C), the distribution curve shifted to the left and displayed a trimodal distribution, including a peak near the submicron (0.01–1 μm) population and two peaks in the micron (1–100 μm) population. The size of the PP particles was represented by their surface area weighted D (3,2) and volume weighted (D (4,3) mean particle diameters (Figure 1b). The D ( 3,2) is most sensitive to fine particulates in the size distribution, while the D (4,3) reflects the size of those particles constituting the bulk of the sample volume [1]. These values decreased appreciably from around 76.1 μm (C sample) to 56.5 μm, 31.1 μm to 10.6 μm, and from around 165.3 μm (C sample) to 103.1 μm, 80.9 μm, and 74.4 μm after OH application at 100 °C, 130 °C, and 150 °C, respectively. The reduction in particle size was directly related to the increase in treatment temperature. These results indicated that OH application, particularly at the highest treatment temperature tested (150 °C), successfully reduced the dimensions of the PP particles into the submicron region.

**Figure 2:**
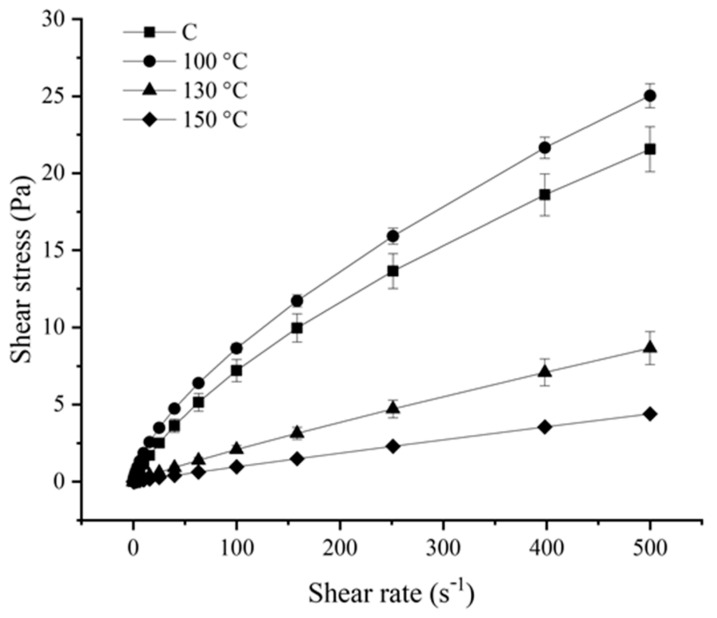
Flow curves of control (C) and ohmic heating (OH)-treated pea protein (PP) dispersions.

Considering the differences observed relative to the particle size distribution of PP dispersion upon OH application, it was also anticipated that distinct rheological properties could also be observable in the OH-treated PP dispersions, compared to the C sample. Therefore, the flow curves of shear stress as a function of shear rate for all samples (including the C sample) were obtained and displayed in Figure 2.

From the observation of the obtained flow curves, it was possible to conclude that OH application, particularly at 130 °C and 150 °C, significantly impacted the flow behaviour of PP dispersion. The C samples displayed a typical pseudoplastic behaviour. A similar flow behaviour was observed for the OH-treated sample at 100 °C. Oppositely, the OH-treated samples at higher temperatures (*ca*. 130 °C and 150 °C) presented an apparent linear behaviour, being more pronounced for the last treatment temperature tested [16]. The fitting parameters displayed in Table 1 confirmed the distinct flow behaviours between the C sample and the OH-treated samples at all treatment temperatures tested.

With the reduction of the particle size, a reduction in viscosity and consistency index was expected [17]. The C sample and the OH-treated sample at 100 °C displayed a distinct behaviour, as observed in Figure 2, that was corroborated by a higher consistency index. At 100 °C, aggregate size reduction was observed, which can have altered aspects such as exposed groups or solvent interactions. In this initial stage of aggregate dissociation, smaller aggregates or individual proteins may have been released in the solvent, maximizing interactions and originating a more structured system. Such structural modifications may have led to a higher resistance to the force applied when compared with the C sample, reflecting an apparent increase in viscosity. For these samples, the *n* value obtained was lower than 1, confirming their pseudoplastic nature. The increase of treatment temperature and aggregate size reduction, led to a significant decrease of the consistency index. Parameter k is related to the dispersion’s viscosity, being significantly lower for the OH-treated samples at 130 °C and 150 °C, indicating viscosity reduction. At the same time, an increase in the *n* value was observed in these samples. The increase in the *n* can be interpreted as the flow behaviour moving towards a Newtonian flow. For the OH-treated sample at 150 °C, the *n* value obtained was close to 1, confirming a more linear behaviour, displaying a behaviour close to a Newtonian fluid.

In commercial PP isolates, as the one used in the present study, both soluble and insoluble aggregates can be formed because of the industrial production of such ingredients, which often compromises their technological–functional properties. We have previously established that OH application at 100 °C caused the dissociation of the existing insoluble macroaggregates/precipitates, and it was also proposed that the application of OH at higher treatment temperatures (i.e., 130 °C and 150 °C) led to the formation of small (soluble) aggregates or peptide fragments [15]. The results, regarding the formation of smaller particles with the increased treatment temperature, are in line with this. Moreover, the dissociation of the large aggregates by the application of OH at 100 °C, could have also contributed to the observed increase of the consistency index (Table 1) compared to the C sample. In fact, the presence of large (dissociated) aggregates could have led to a greater resistance to the applied force, contributing to the increase in the viscosity of this sample. The presence of small (soluble) aggregates or peptide fragments in the OH-treated samples at 130 °C and 150 °C possibly offered less resistance to the applied force, resulting in the observed lower viscosity of these samples and the displayed linear behaviour of the last sample, in agreement with a Newtonian fluid.

### 2.2. Foaming and Emulsifying Properties

**Figure 3:**
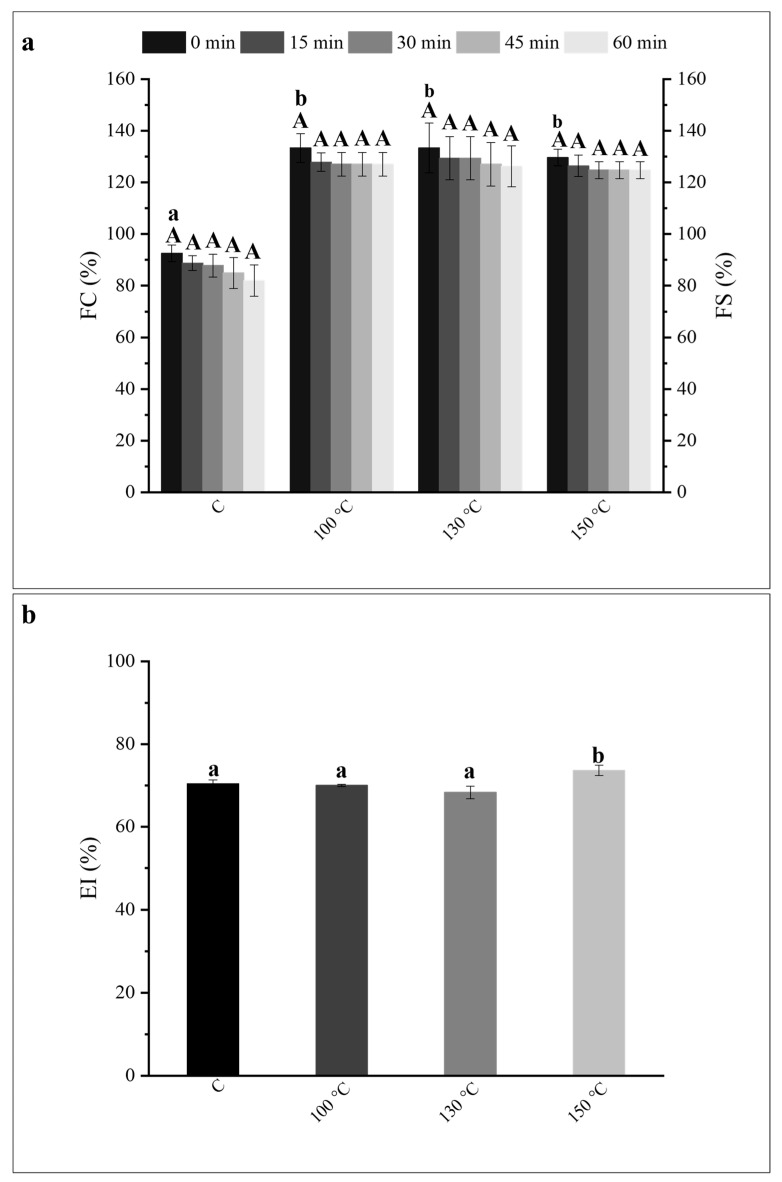
(**a**) Foaming capacity (FC) and foam stability (FS) of control (C) and ohmic heating (OH)- treated pea protein (PP) dispersions. (**b**) Emulsion index of C and OH-treated PP dispersions, after 7 days. (**a**) For each column, different lower-case letters correspond to statistically significant differences (*p* < 0.05) in the FC (0 min) between all conditions tested (including C) and different capital letters correspond to statistically significant differences (*p* < 0.05) in the FS (15 min, 30 min, 45 min and 60 min) of each sample (including C).

The FC and foaming stability (FS) of OH-treated PP dispersions at 100 °C, 130 °C, and 150 °C are presented in Figure 3a.

OH treatments in all tested temperatures—i.e., 100 °C, 130 °C, and 150 °C—resulted in an increase of the FC of PP dispersions, corresponding to increases of 44%, 44%, and 40%, respectively, when compared to the C sample. No significant differences were observed between the OH-treated samples, suggesting a similar impact of all OH treatments in the improvement of the FC of commercial PP. No significant (*p* < 0.05) differences were observed regarding the FS of all OH treatments, indicating that all produced foams remained stable during the determined period (60 min), including for the C sample. It is established that proteins adsorb at the gas–water surface, reducing the surface tension. The hydrophobic groups of proteins position towards the gas phase, while hydrophilic groups are exposed to the continuous liquid phase. This leads to the formation of a flexible, cohesive, and mechanically strong film surrounding the gas bubbles due to protein–protein interactions, providing stability against foam destabilization mechanisms such as coarsening, drainage, and coalescence [18]. In this sense, and according to what was previously referred, the dissociation of insoluble macroaggregates/precipitates with consequent size reduction and exposure of hydrophobic groups upon OH application, led to the enhancement of commercial PP’s foaming performance [19]. Interesting to notice that despite the recent findings [15] demonstrating a distinct impact of the different OH treatments applied on PP’s structure and solubility, the same tendency was not observed regarding the FC of PP. Indeed, the application of OH at increased treatment temperatures resulted in an equal improvement of this functional property.

Regarding the emulsifying ability of commercial PP upon OH application, the emulsion index (EI) values of the C and OH-treated samples after 7 days are displayed in Figure 3b. All the emulsions formed (including the C sample) remained stable after 7 days, and no differences were observed between those and the ones analyzed on day 1. Despite the observation of a small but significant (*p* < 0.05), increase of 4% in the OH-treated sample at 150 °C, overall, the OH application at all treatment temperatures applied did not lead to a noticeable improvement of the emulsifying properties of the commercial PP despite the positive impact of OH at the selected temperatures in improving PP solubility. This was somewhat surprising, considering the changes observed on the other properties and given the known impact of denaturation and aggregation in protein emulsifying properties [20].

The here obtained results demonstrate that the foaming and emulsifying attributes of PP are influenced by a complex set of factors (including intrinsic protein characteristics such as molecular weight, conformation, water solubility, amino acid composition, and hydrophobicity–hydrophilicity ratio at the surface and external parameters such as pH, temperature, and ionic strength), that interact with each other and interfere with the interfacial activity of PP [21]. Such distinct behaviour could be attributed to the ability of the resulting smaller aggregates and peptide fragments in providing rapid adsorption at the air–water interface (thus improving foaming capacity) but failing in forming a sufficiently strong, flexible, viscous film at the oil–water interface to prevent droplet amalgamation (thus limiting emulsifying ability). However, a more in-depth study is required to better explore how the combination of these factors can result in improved foaming and/or emulsifying performances of commercial PP ingredients.

### 2.3. Cold-Set Gels Appearance and Viscoelastic Properties

**Figure 4:**
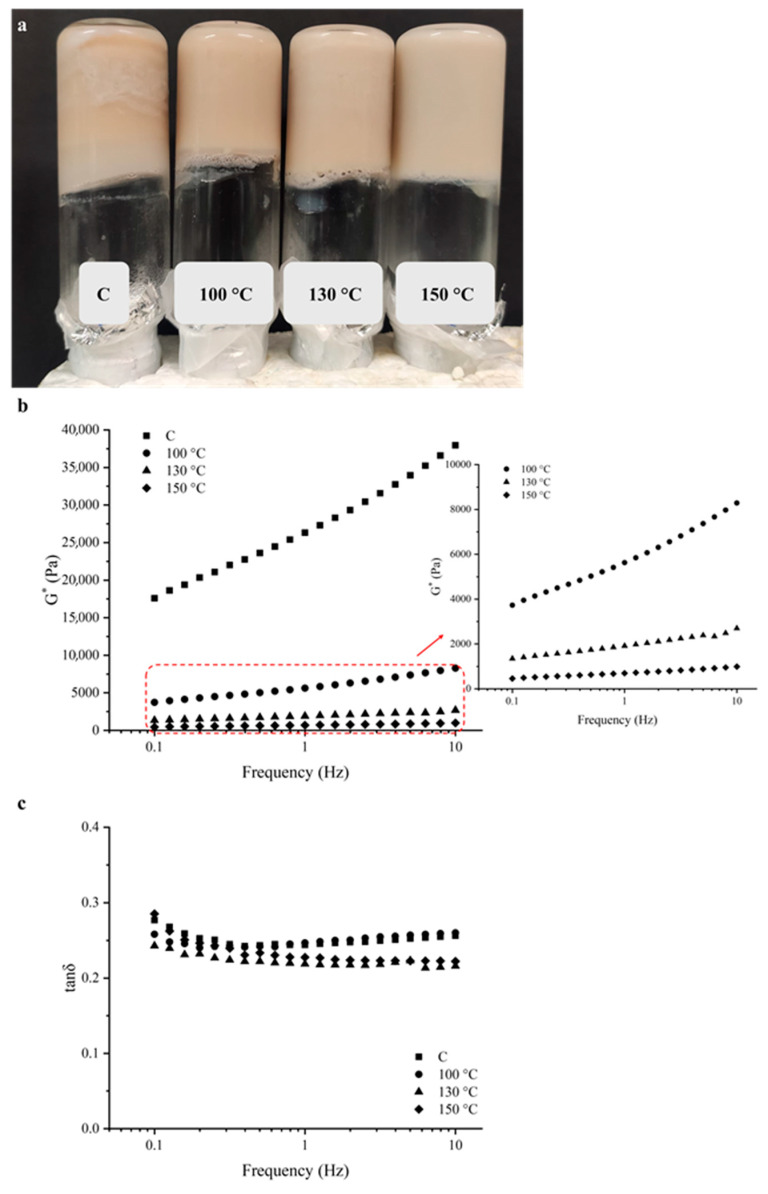
(**a**) Appearance, frequency dependence of (**b**) complex (G*) modulus and (**c**) tan *δ* of the cold-set pea protein (PP) gels prepared from control (C) and ohmic heating (OH)-treated pea protein (PP) dispersions.

In Figure 4a, the obtained gels on inverted vials are presented. Through this simple method, it was possible to verify the gel formation by the absence of material’s flow. The control sample (C) displayed partial phase separation and heterogenic, indicating a possible weak gel formation and poor structural integrity. The gels obtained by OH-treated samples at 100 °C, although to a lower extent, which also displayed some heterogenicity and minor phase separation, while, in contrast, the gels obtained by samples treated at 130 °C and 150 °C appeared more homogeneous and opaquer, suggesting the development of a more compact and continuous network structure. Protein aggregation and size distribution are known to influence the gel properties, and larger protein aggregates originate coarser network structures [22]. Rheological tests were performed for all samples to confirm the cold-set gel formation and to assess their viscoelastic properties and revealed that the gel properties were strongly influenced by the processing conditions. Frequency sweeps of complex (G*) modulus and tan *δ* were carried out in the viscoelastic linear region (Figure 4b,c).

The viscoelastic behaviour of the produced cold-set gels was characterized by the prevalence of the elastic over the viscous component (*G*′ > *G*″), thus confirming the formation of a gel in all tested samples. However, a clear differentiation between the gels obtained from the C and the OH-treated samples was observed, with the higher values of G* for the C sample, but with a substantial increase with the increase in angular frequency. A gel such as C, with higher G* but stronger frequency dependence is stiffer yet less stable—it behaves elastically under rapid deformation but relaxes under slow deformation. This indicates a transient or weakly crosslinked network rather than a fully developed, frequency-independent elastic gel. For OH-treated samples, this behaviour becomes less pronounced, with the OH application at increased treatment temperatures (i.e., 100 °C, 130 °C, and 150 °C). Usually, the viscoelastic moduli do not show frequency dependency in stronger or well-structured gels originating from chemical crosslinking. In contrast, weaker or less structured gels, dependent on physical interactions, viscoelastic moduli display great frequency dependency. The small magnitude and low dependency of G* on the frequency are typical of a soft gel, formed by a combination of chemical crosslinking and physical interactions [23]. Figure 4c displays the variation of tan *δ* with the frequency sweep. The determined tan *δ* values were similar for all the cold-set gels obtained. In general, all samples displayed tan *δ* values lower than 1 but higher than 0.1 with barely any frequency dependence, which is consistent with a weak gel behaviour [14,24,25].

It is interesting to notice that, despite the proximity to the other samples, the cold-set gels obtained from the C sample and the OH-treated sample at 100 °C displayed higher tan *δ* values with the tendency to increase at higher frequencies. This is indicative of an increase of the viscous component or a possible unstable network, which is compatible with a heterogeneous structure, in agreement with the visual observation of the obtained gels, particularly for the C sample, which displays a heterogeneous appearance (Figure 4a). The analysis of the viscoelastic moduli at the reference frequency of 1 Hz was performed and is displayed in Table 2.

The data analysis demonstrated that significant differences induced by OH application at all treatment temperatures occurred. As observed in Figure 4b, G* was significantly higher for the C sample, revealing a stiffer gel. Even though no significant (*p* < 0.05) differences were observed between the OH-treated samples, a progressive decrease was detected, resulting in the differentiation of the gel strength. As previously reported, the presence of insoluble macroaggregates is considered unfavourable for the formation of a well-structured gel network, forming a more coagulated structure [26]. Moreover, according to Shand et al., the number of cross-links within the gelled network increases with the soluble pea protein aggregates available, directly dependent on the heat treatment [27]. These results, in line with the previously referred, confirm the positive impact of OH application, particularly at higher temperatures (i.e., 130 °C and 150 °C), to the formation of soft gels but with a more compact network. Nevertheless, further investigation should be performed to optimize the conditions of cold-set gel formulation depending on the intended food application.

### 2.4. Cold-Set Gels Microstructure

**Figure 5:**
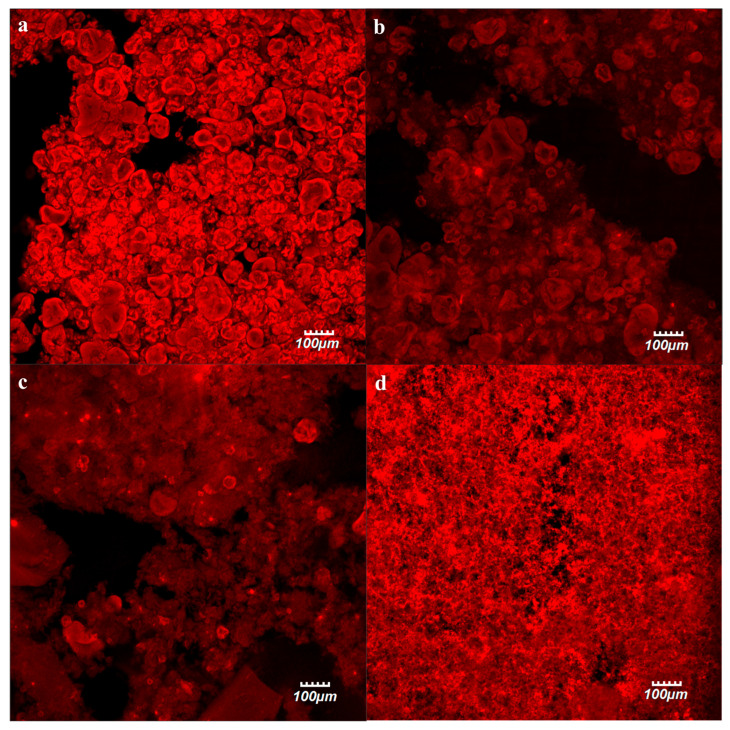
Confocal microscopy images of pea protein (PP) cold-set gels, with the protein stained in red: (**a**) control (C) and ohmic heating (OH)-treated at: (**b**) 100 °C, (**c**) 130 °C, and (**d**) 150 °C.

There is a strong relation between the microstructure and rheology of gels formed by aggregated protein particles [28]. In order to elucidate these relations, the microstructure of PP cold-set gels submitted to distinct OH treatments at 100 °C, 130 °C, and 150 °C was investigated by confocal laser scanning microscopy (CLSM) as displayed in Figure 5.

The gels obtained from the C samples (Figure 5a) displayed a network mainly composed of large aggregates. For the remaining samples (Figure 5b,c), a progressive reduction in the amount of these large aggregates was observed, becoming more pronounced with the increase in the treatment temperature. Cold-set gels that were obtained from the OH-treated samples at 100 °C (Figure 5b) displayed a similar structure to that of those C samples, composed by large aggregates, but now connected by a fine network. In the samples, they were obtained from OH-treated samples at 130 °C (Figure 5c), although some large aggregates are still observable, the fine network was predominant. And finally, the samples OH-treated at 150 °C (Figure 5d) displayed a uniform appearance with the visualization of a fine protein network and the absence of the large protein aggregates previously detected. These results corroborate the visual observations and rheological determinations, where sample C is composed of large, poorly dispersed aggregates, which indicates that its mechanical properties are dominated by weak physical contacts among large particles rather than by a continuous viscoelastic network. OH treatments promoted substantial particle reduction and protein dissolution, along with molecular-level reorganization. These factors resulted on a homogeneous, continuous matrix. These findings demonstrated how particle refinement and solubilization enhance interparticle connectivity and viscoelastic solid behavior, culminating in a thermally consolidated network capable of sustaining stress over long timescales.

OH effectively reduced the size of particles present in the PP dispersions, directly interfering with its viscosity and flow behaviour. The structural rearrangements occurring on the commercial PP upon OH application led to a significant improvement of its foaming and gelling properties. Through these findings, this study contributes to the growing body of research on physical and electric-field-assisted modification of plant proteins, demonstrating how ohmic heating can be used to tailor the structure–function relationships of commercial pea protein. Unlike conventional thermal or mechanical treatments, OH enables controlled structural rearrangements that translate into measurable functional outcomes, particularly in foaming and gelation behavior.

Nevertheless, the absence of a significant enhancement of the emulsifying properties reinforces the challenge of modifying this functional property. As already referred, the proteins’ ability to act as emulsifiers is highly influenced by a complex set of factors, both intrinsic to proteins and also external parameters, that interact with each other and interfere with their interfacial activity. Thus, further investigation is needed to fully harness the potential advantage of the structural rearrangements occurring on PP upon OH application.

OH application highly interfered with the gelling ability of PP, demonstrating that the formation of soluble aggregates from insoluble ones indeed contributed to the development of more uniform and fine-stranded gelled networks, which was confirmed by the microstructure analysis. In a broader context, this work supports the development of sustainable and energy-efficient food processing technologies aligned with policy goals promoting plant-based protein consumption. By improving the functional performance of pea protein ingredients, OH processing can contribute to the development of plant-based foods with improved texture and quality, ultimately benefiting consumers and facilitating wider acceptance of sustainable dietary alternatives.

## 3. Conclusions

Ohmic heating (OH) effectively modified the structure and functionality of commercial pea protein. OH treatments at 100–150 °C reduced particle size, with D (3,2) decreasing from ≈76 µm in the untreated sample to ≈11 µm at 150 °C, indicating disruption of insoluble aggregates. These changes resulted in a significant increase in foaming capacity (≈40%), while foam stability remained unchanged over 60 min. Emulsifying activity was largely unaffected, showing only a minor increase at 150 °C.

Cold-set gels were obtained from all samples tested, either untreated (C sample) or after OH application, at all conditions tested. The obtained gels from the treated samples displayed distinctive rheological properties from those obtained from the unprocessed PP. Indeed, rheological analysis of the gels obtained from the unprocessed PP revealed a possible unstable or weakened structure, while the gels obtained from treated samples displayed a soft gel behaviour, but more homogeneous gel networks, as supported by rheological and CLSM analyses. The results obtainable on this study established the potential of OH to be used as an effective tool for developing PP systems with tailored functionality. Despite the current findings, continued research is essential to clarify the extent to which it may influence the interfacial activities of these proteins and ultimately their functional role in emulsification.

## 4. Materials and Methods

### 4.1. Materials

Pea protein (PP) isolate was kindly supplied by Cosucra (COSUCRA Groupe Warcoing S.A., Pecq, Belgium. Calcium chloride was supplied by Sigma-Aldrich (Steinheim, Germany) and was used to induce cold-set gelation of PP. All other reagents used were of analytical grade.

### 4.2. Sample Preparation

PP dispersions at 10% (*w*/*v*) were prepared in sodium phosphate buffer (0.01 mol·L^−1^ at pH 7). The dispersions were magnetically stirred at room temperature for 2 h and, if needed, pH was adjusted to 7 with 1 mol·L^−1^ hydrochloric acid or sodium hydroxide. The PP dispersions were then stored overnight at 4 °C and submitted on the next day to ohmic heating (OH). Unprocessed PP dispersions were used as control (C) samples.

### 4.3. OH Processing of PP Dispersions

OH treatments were conducted in triplicate, on an air-thigh glass reactor. Two stainless steel electrodes were positioned at each extremity of the glass reactor and isolated by polytetrafluoroethylene caps, as described elsewhere [15]. The OH reactor was connected to a power supply generating an electric signal (20 kHz and an electric field strength of 25 V·cm^−1^), allowing the samples to be heated until reaching the target treatment temperature of 100 °C, 130 °C, and 150 °C. Homogeneity of the sample was ensured using a magnetic stirrer inside the reactor. Once the target temperature as reached, the power was shut down and samples were let to cool down to room temperature, transferred to screw cap tubes and placed on an ice bath for 15 min. Part of the solutions was collected and diluted to 1% (*w*/*v*) for the determination of foaming and emulsifying properties, and the remaining was used in particle size, rheological, microstructure analysis, and in gelation experiments.

### 4.4. Gel Preparation

Cold-set gels were prepared with CaCl_2_ by adding 5 mol·L^−1^ solution to the protein dispersions until a final concentration of 0.02 mol·L^−1^. The mixture was homogenized on a vortex mixer and let set for 24 h at 20 °C before rheological analysis [29].

### 4.5. Characterization of PP Dispersions upon OH Processing

#### 4.5.1. Particle Size Analysis

To determine the particle size distribution and mean particle diameters of PP dispersions, a Malvern Mastersizer 3000 laser diffraction instrument equipped with a Hydro MV sample dispersion unit (Malvern Panalytical, Malvern, UK). PP dispersions at 10% (*w*/*v*) were fed to the sample dispersion unit prefilled with distilled water. Measurements were initiated once an obscuration level between 2% and 8% was obtained, as recommended by the manufacturer. The stirring of the dispersion unit was 2400 rpm, the refractive indices were set at 1.33 and 1.47, for water and protein particles, respectively. The particle size distribution and mean particle diameters (D(3,2) and D(4,3)) of the samples were recorded.

#### 4.5.2. Rheological Analysis

Rheological measurements, determined in triplicate, at 25 °C in a TA Instruments HR- 1 rheometer equipped with a Peltier plate (TA Instruments, New Castle, DE, USA). Flow curves were obtained for the PP dispersions at 10% (*w*/*v*) using a cone-plate (60 mm, 2° angle, truncation gap of 64 μm) through a three-step program (up-down-up) using a continuous ramp and shear rate range from 0.1 to 500 s^−1^. The three-step program was performed to eliminate the time-dependence. The flow behaviour of the selected PP dispersions was characterized by the power-law model (Equation (1)) that was fitted to the experimental data obtained:

(1) $$\sigma =k\times {\overset{\cdot }{\gamma }}^{n}$$

where σ represents the shear stress (Pa), $\overset{\cdot }{\gamma }$ is the shear rate (*s^−1^*), *k* is consistency index (Pa.s−1), and *n* is power-law index (i.e., *n* = 1 for Newtonian, *n* < 1 for pseudoplastic, and *n* > 1 for dilatant fluids).

### 4.6. Determination of Functional Properties of OH-Treated PP Dispersions

#### 4.6.1. Foaming Capacity and Foam Stability

The foaming capacity (*FC*) and foam stability (*FS*) were determined by pouring 30 mL of 1% (*w*/*v*) PP dispersions, followed by mixing with a handheld milk foamer (with 14,000 rpm high-speed motor inside) for 1 min, at RT. The volume of the fluid before (*V1*) and fluid+ foam after (*V2*) homogenization was recorded [30]. The total volume (*Vt*) was recorded periodically (15 min, 30 min, 45 min, and 60 min), while the sample was kept at room temperature. FC (Equation (2)) and FS (Equation (3)) were calculated as follows:

(2) $$FC\;\left(\%\right)=\;\frac{V_{2}-V_{1}}{V_{1}}\;\times 100$$

(3) $$FS\;\left(\%\right)=\frac{V_{t}-V_{2}}{V_{2}}\times 100$$

#### 4.6.2. Emulsion Index

The emulsifying capacity of protein solutions can be assessed through the determination of the emulsion index (EI) [31]. For that, 5 mL of 1% (*w*/*v*) OH-treated PP dispersions were mixed with 5 mL of sunflower oil and then stirred for 2 min using a vortex mixer at 3500 rpm, leaving it to stand for 1 and 7 days. The EI (%) was then calculated as the percentage of emulsified layer height (Ae) divided by total height (At) of the liquid column, as follows:

(4) $$EI\;\left(\%\right)=\;\frac{Ae}{At}\;\times 100$$

#### 4.6.3. Rheological Characterization of PP Cold-Set Gels

Oscilattory rheology was used to determine the viscoelastic properties of the PP cold-set. Determinations were performed, in triplicate, at 25 °C in a TA Instruments HR- 1 rheometer equipped with a Peltier plate (TA Instruments, New Castle, DE, USA). The frequency sweep tests were performed between 0.1 and 10 Hz, using a plate-plate geometry (40 mm, 500 μm gap), within the linear viscoelasticity domain (0.5% strain). The complex (G*) modulus and tan δ (G″/G′) were evaluated [14].

#### 4.6.4. Microstructure Study of PP Cold-Set Gels

The microstructure of PP cold-set gels was observed through Confocal Laser Scanning Microscopy (CLSM). Samples were prepared as previously described by Rodrigues et al., [14]. In brief, OH-treated samples were stained with rhodamine B isothiocyanate and the cold-set gelation procedure was performed. Stained PP cold-set gels were then placed on a microscope slide, covered with a slip, and hermetically sealed to avoid evaporation. A Confocal Scanning Laser Microscope (Olympus BX61, Model FluoView 1000 Version 4.2.1.20) was used to analyze the microstructure, in fluorescent mode (laser excitation line 559 nm).

### 4.7. Statistical Analysis

Statistical analyses were performed on the OriginPro (version 9.5, 2018) software from OriginLab Corporation (Northampton, MA, USA). Significant differences based on at least three individual measurements were determined by a one-way ANOVA and Tukey’s test for multiple comparisons using a *p-value* of 0.05. Unless otherwise stated, all treatments were repeated at least three times using a different batch of samples.

## Figures and Tables

**Table 1:** Power-law fitted parameters to the flow curves.

	Parameters	
Sample	*k* (Pa.s^−1^)	*n*
C	0.290 ± 0.056 ^a^	0.697 ± 0.020 ^a^
100 °C	0.420 ± 0.018 ^b^	0.658 ± 0.002 ^b^
130 °C	0.036 ± 0.005 ^c^	0.883 ± 0.009 ^c^
150 °C	0.013 ± 0.000 ^c^	0.942 ± 0.000 ^d^

For each column, different letters correspond to statistically significant differences (*p* < 0.05). All correlation coefficients of model fitting to experimental data were >0.99.

**Table 2:** Complex (G*) modulus and tan *δ* measured at 1 Hz.

Sample	G* (Pa)	tan *δ*
C	25,161.93 ± 12,246.96 ^a^	0.244 ± 0.003 ^a^
100 °C	5417.54 ± 1069.60 ^b^	0.277 ± 0.049 ^a^
130 °C	1987.21 ± 216.12 ^b^	0.238 ± 0.025 ^a^
150 °C	839.53 ± 226.84 ^b^	0.223 ± 0.006 ^a^

For each column, different letters correspond to statistically significant differences (*p* < 0.05).

## Data Availability

The original contributions presented in this study are included in the article. Further inquiries can be directed to the corresponding author.

## Abbreviations

The following abbreviations are used in this manuscript:

C	control
CLSM	Confocal laser scanning microscopy
EI	Emulsion index
FC	Foaming capacity
FS	Foaming stability
MEF	Moderate electric fields
OH	ohmic heating
PP	Pea protein

## References

[1] Ren W., Xia W., Gunes D.Z., Ahrné L. (2024). Heat-induced gels from pea protein soluble colloidal aggregates: Effect of calcium addition or pH adjustment on gelation behavior and rheological properties. Food Hydrocoll..

[2] Shanthakumar P., Klepacka J., Bains A., Chawla P., Dhull S.B., Najda A. (2022). The Current Situation of Pea Protein and Its Application in the Food Industry. Molecules.

[3] Lam A.C.Y., Karaca A.C., Tyler R.T., Nickerson M.T. (2018). Pea protein isolates: Structure, extraction, and functionality. Food Rev. Int..

[4] Burger T.G., Singh I., Mayfield C., Baumert J.L., Zhang Y. (2022). Comparison of physicochemical and emulsifying properties of commercial pea protein powders. J. Sci. Food Agric..

[5] Mession J.L.L., Chihi M.L., Sok N., Saurel R. (2015). Effect of globular pea proteins fractionation on their heat-induced aggregation and acid cold-set gelation. Food Hydrocoll..

[6] Li C., McClements D.J., Dai T., Deng L., Feng Z., Li T., Liu C., Chen J. (2023). Enhancing the dispersibility of commercial pea protein ingredients using stirred media milling: Potential mechanisms of action. Food Hydrocoll..

[7] Moll P., Salminen H., Griesshaber E., Schmitt C., Weiss J. (2022). Homogenization improves foaming properties of insoluble pea proteins. J. Food Sci..

[8] Cui L., Bandillo N., Wang Y., Ohm J.-B., Chen B., Rao J. (2020). Functionality and structure of yellow pea protein isolate as affected by cultivars and extraction pH. Food Hydrocoll..

[9] Goyal N., Thakur R., Yadav B.K. (2024). Physical Approaches for Modification of Vegan Protein Sources: A Review. Food Bioprocess Technol..

[10] Wu D., Wu C., Wang Z., Fan F., Chen H., Ma W., Du M. (2019). Effects of high pressure homogenize treatment on the physicochemical and emulsifying properties of proteins from scallop (*Chlamys farreri*). Food Hydrocoll..

[11] Avelar Z., Vicente A.A., Saraiva J.A., Rodrigues R.M. (2021). The role of emergent processing technologies in tailoring plant protein functionality: New insights. Trends Food Sci. Technol..

[12] Rodrigues R.M., Vicente A.A., Petersen S.B., Pereira R.N. (2019). Electric field effects on β-lactoglobulin thermal unfolding as a function of pH—Impact on protein functionality. Innov. Food Sci. Emerg. Technol..

[13] Chen Y., Wang T., Zhang Y., Yang X., Du J., Yu D., Xie F. (2022). Effect of moderate electric fields on the structural and gelation properties of pea protein isolate. Innov. Food Sci. Emerg. Technol..

[14] Rodrigues R.M., Fasolin L.H., Avelar Z., Petersen S.B., Vicente A.A., Pereira R.N. (2020). Effects of moderate electric fields on cold-set gelation of whey proteins—From molecular interactions to functional properties. Food Hydrocoll..

[15] Avelar Z., Saraiva J.A., Vicente A.A., Rodrigues R.M. (2024). Unravelling the impact of ohmic heating on commercial pea protein structure. Food Hydrocoll..

[16] Steffe J.F. (1996). Rheological Methods in Food Process Engineering.

[17] Nicoud L., Lattuada M., Yates A., Morbidelli M. (2015). Impact of aggregate formation on the viscosity of protein solutions. Soft Matter.

[18] Amagliani L., Silva J.V.C., Saffon M., Dombrowski J. (2021). On the foaming properties of plant proteins: Current status and future opportunities. Trends Food Sci. Technol..

[19] Liu X., Wei K., Wang Z., Jiang L., Du J., Huang Z., Tian T. (2025). Structural modification and foaming enhancement of winged bean protein isolate via heat treatment: Implications for high-quality plant-based whipped cream applications. Food Hydrocoll..

[20] Palazolo G.G., Sorgentini D.A., Wagner J.R. (2004). Emulsifying properties and surface behavior of native and denatured whey soy proteins in comparison with other proteins. Creaming stability of oil-in-water emulsions. J. Am. Oil Chem. Soc..

[21] Queirós R., Ferreira R., Saraiva J.A., Lopes-da-Silva J.A. (2023). High-Pressure Effects on Selected Properties of Pea and Soy Protein Isolates. Appl. Sci..

[22] Klost M., Brzeski C., Drusch S. (2020). Effect of protein aggregation on rheological properties of pea protein gels. Food Hydrocoll..

[23] Ross-Murphy S.B. (1995). Structure–property relationships in food biopolymer gels and solutions. J. Rheol..

[24] Gonçalves R.F.S., Zhou H., Vicente A.A., Pinheiro A.C., McClements D.J. (2023). Plant-based bigels for delivery of bioactive compounds: Influence of hydrogel:oleogel ratio and protein concentration on their physicochemical properties. Food Hydrocoll..

[25] Savadkoohi S., Farahnaky A. (2012). Dynamic rheological and thermal study of the heat-induced gelation of tomato-seed proteins. J. Food Eng..

[26] Sun X.D., Arntfield S.D. (2010). Gelation properties of salt-extracted pea protein induced by heat treatment. Food Res. Int..

[27] Shand P.J., Ya H., Pietrasik Z., Wanasundara P. (2007). Physicochemical and textural properties of heat-induced pea protein isolate gels. Food Chem..

[28] van Vliet T. (2000). Structure and rheology of gels formed by aggregated protein particles. Hydrocolloids.

[29] Ju Z.Y., Kilara A. (1998). Aggregation Induced by Calcium Chloride and Subsequent Thermal Gelation of Whey Protein Isolate. J. Dairy Sci..

[30] Shevkani K., Singh N., Kaur A., Rana J.C. (2015). Structural and functional characterization of kidney bean and field pea protein isolates: A comparative study. Food Hydrocoll..

[31] Li X.-M., Xie Q.-T., Zhu J., Pan Y., Meng R., Zhang B., Chen H.-Q., Jin Z.-Y. (2019). Chitosan hydrochloride/carboxymethyl starch complex nanogels as novel Pickering stabilizers: Physical stability and rheological properties. Food Hydrocoll..

